# Interleukin 10 inhibits pro-inflammatory cytokine responses and killing of *Burkholderia pseudomallei*

**DOI:** 10.1038/srep42791

**Published:** 2017-02-20

**Authors:** Bianca Kessler, Darawan Rinchai, Chidchamai Kewcharoenwong, Arnone Nithichanon, Rachael Biggart, Catherine M. Hawrylowicz, Gregory J. Bancroft, Ganjana Lertmemongkolchai

**Affiliations:** 1 Centre for Research and Development of Medical Diagnostic Laboratories, Khon Kaen University, Khon Kaen, Thailand; 2 Department of Immunology and Infection, London School of Hygiene & Tropical Medicine, London, United Kingdom; 3 Department of Asthma, Allergy and Respiratory Science, Guys Hospital, Kings College, London, United Kingdom

## Abstract

Melioidosis, caused by *Burkholderia pseudomallei*, is endemic in northeastern Thailand and Northern Australia. Severe septicemic melioidosis is associated with high levels of pro-inflammatory cytokines and is correlated with poor clinical outcomes. IL-10 is an immunoregulatory cytokine, which in other infections can control the expression of pro-inflammatory cytokines, but its role in melioidosis has not been addressed. Here, whole blood of healthy seropositive individuals (n = 75), living in N. E. Thailand was co-cultured with *B. pseudomallei* and production of IL-10 and IFN-γ detected and the cellular sources identified. CD3^−^ CD14^+^ monocytes were the main source of IL-10. Neutralization of IL-10 increased IFN-γ, IL-6 and TNF-α production and improved bacteria killing. IFN-γ production and microbicidal activity were impaired in individuals with diabetes mellitus (DM). In contrast, IL-10 production was unimpaired in individuals with DM, resulting in an IL-10 dominant cytokine balance. Neutralization of IL-10 restored the IFN-γ response of individuals with DM to similar levels observed in healthy individuals and improved killing of *B. pseudomallei in vitro*. These results demonstrate that monocyte derived IL-10 acts to inhibit potentially protective cell mediated immune responses against *B. pseudomallei,* but may also moderate the pathological effects of excessive cytokine production during sepsis.

*Burkholderia pseudomallei (B. pseudomallei*) is a gram-negative bacterium that causes melioidosis. It is highly endemic in northeast Thailand and Northern Australia. However there are increasing reports in other regions such as South America, India and Malaysia^1^,^2^. In endemic areas, *B. pseudomallei* is isolated from soil, stagnant water and rice paddies^3^. Several clinical outcomes ranging from asymptomatic to localized infection, to fatal acute septicemia are associated with melioidosis^4^ and relapse of disease could still occur after infection and treatment^3^. Melioidosis is a major cause of septicemia-associated deaths in N.E. Thailand, with mortality rates of up to 40% even with current best-practice clinical management^5^.

Several risk factors have been associated with susceptibility to melioidosis, but diabetes mellitus (DM) is the most important, and reported in 60% of melioidosis patients^6^. The increased susceptibility to infection in individuals with DM has been attributed to reduced IL-12 and interferon gamma (IFN-γ) production by monocytes and natural killer (NK) cells/T cells respectively^7^ in response to *B. pseudomallei*. Polymorphonuclear leukocyte (PMNs) of Thai individuals with DM also have reduced pro-inflammatory cytokine production^8^, and other functions including phagocytosis, migration and apoptosis are also impaired^9^. PMNs also play an important role in *B. pseudomallei* infection in experimental models, suggesting that together, these PMNs defects are likely to contribute to the increased risk to melioidosis in individuals with DM^10^.

Given the spectrum of disease manifestations *B. pseudomallei* provokes, it is likely that the outcome of infection will be critically influenced by the balance between potentially protective pro-inflammatory responses which promote bacterial killing versus the risks of immune pathology and septic shock. Studies in mice have found that cytokines such as IFN-γ in particular but also other pro-inflammatory cytokines such as IL-12, tumor necrosis factor (TNF-α), and IL-18 are essential for resistance against *B. pseudomallei* since depletion of these cytokines *in vivo*, decreases survival and increases blood and tissue bacterial burden^11^,^12^,^13^,^14^,^15^. In both mice and humans, NK, CD4^+^ and CD8^+^ T cells are the cellular sources of IFN-γ in response to *B. pseudomallei*^16^,^17^,^18^,^19^. However, in other systems, these and other cytokines can also have a detrimental role by promoting the development of septic shock^20^. In the context of melioidosis, IFN-γ and its inducing cytokines, IL-18, IL-12 and IL-15 along with TNF-α and IL-6 have shown to correlate with disease severity in melioidosis patients^21^,^22^. To protect against immune-pathology, anti-inflammatory responses (such as T helper (Th) 2/regulatory T cells (Treg) and others) are crucial to control excessive pro-inflammatory cytokine cascades but to date their role in melioidosis has not been addressed.

IL-10 is a potent anti-inflammatory immunosuppressive cytokine with a broad range of effects both directly and indirectly on innate and adaptive immunity^23^. It is important in dampening inflammatory responses but can contribute to pathogen persistence^24^. Several cell types can produce IL-10 but for many infections the most important *in vivo* sources are monocytes, macrophages, Tregs, Th2 T cells^25^ and other CD4^+^ T cells that produce both IL-10 and IFN-γ^26^. In particular, macrophages produce IL-10 in a negative and positive feedback loop to dampen the uncontrolled inflammatory cytokine production during infection. IL-10 expression is tightly controlled by IFN-γ and IL-10 itself; it can limit its own expression or positively feedback to amplify its own production. In addition, IFN-γ can interfere with the IL-10 production pathway and block its production through phosphoinositide 3-kinase (PI3K)^27^. Increased IL-10 during infection down regulates many processes including IFN-γ production, which in turn reduces macrophage activation and disrupts the effective cellular response to clear the pathogen^28^. For example, in the absence of IL-10, mice infected with *Mycobacterium tuberculosis* have lower bacterial burdens and also improved protection when vaccinated with BCG^29^,^30^. However, the biology of IL-10 production and action in humans in the context of melioidosis is poorly understood. In acute infection with *B. pseudomallei*, IL-10 levels in plasma are clearly increased, and are greatest in non surviving patients^22^. *B. pseudomallei* replicates within monocytes/macrophages and induces antigen specific T cell responses, but to date the cellular source and function of IL-10 has not been investigated.

In this study we investigated IL-10 cytokine responses to *B. pseudomallei* in cells from individuals living in the melioidosis endemic area in northeast Thailand. Our objectives were to investigate the major cellular sources of IL-10, its potential role in the regulation of cytokine production and killing of *B. pseudomallei* and to determine whether altered IL-10 responses contribute to the increased risk of melioidosis in individuals with DM.

## Results

### *B. pseudomallei* induces IL-10 and IFN-γ production in individuals living in melioidosis endemic area

**Figure 1:**
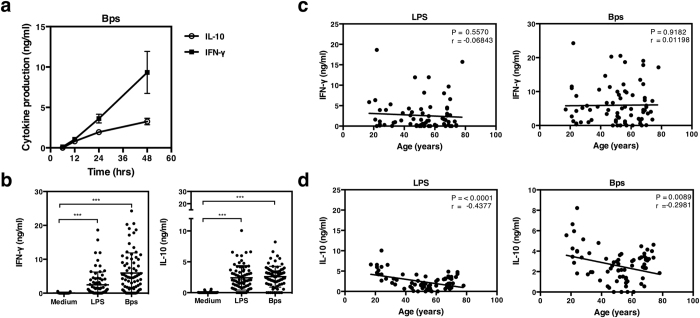
IL-10 and IFN-γ production by whole blood of healthy individuals in response to *B. pseudomallei*. Whole blood of a healthy representative seropositive donor was incubated with killed *B. pseudomallei* (30:1 ratio) for 6, 12, 24 or 48 hours *in vitro* and supernatants assayed for IFN-γ and IL-10 (**a**). Whole blood of healthy seropositive donors (n = 75) was stimulated with killed *B. pseudomallei* (30:1 ratio) or 10 μg/ml of *E. coli* LPS and supernatants assayed for IFN-γ and IL-10 by ELISA after 48 hours *in vitro* (**b**). IFN-γ (**c**) and IL-10 (**d**) production in response to *E. coli* LPS or killed *B. pseudomallei* (30:1 ratio) from experiment performed in (**b**) were plotted according to the age of the individuals. The data in (**b**) are presented with the mean and the standard deviation. Statistical significance was determined using one way ANOVA and Tukey’s post test or Pearson correlation; ns, non significant, *p < 0.05, **p < 0.01 and ***p < 0.001.

We have previously shown that incubation of blood from healthy individuals from the melioidosis endemic region of northeast Thailand with *B. pseudomallei* results in production of pro-inflammatory cytokines including TNF-α, IL-6 and in particular IFN-γ *in vitro*^17^. To now compare the balance between pro and anti-inflammatory cytokine production, whole blood of a healthy representative seropositive donor was stimulated with killed *B. pseudomallei* or lipopolysaccharide (LPS) (a known inducer of IL-10) and 48 hours later assayed for IL-10 and IFN-γ production by ELISA. IL-10 and IFN-γ were produced in response to killed *B. pseudomallei* in a dose dependent manner (data not shown), with similar kinetics, which was maximal at 48 hours of culture (Fig. 1a). When we extended this analysis to a larger donor cohort, 53 and 62 out of 75 individuals responded significantly to *B. pseudomallei* by producing IL-10 and IFN-γ respectively, above medium control (Fig. 1b; *p* < 0.001). IL-10 from 32 individuals and IFN-γ from 53 individuals also responded to LPS above medium control (Fig. 1b; *p* < 0.001). Other studies have shown there is an increased pro-inflammatory response to cytomegalovirus (CMV), Epstein-Barr virus (EMV) and Influenza^31^ with age, which may increase susceptibility to these infections. To determine if age affected the cytokine response to *B. pseudomallei*, we stratified the responses of the 75 donors according to age (with a range from 17 to 78 years old). We found IL-10 production in response to *B. pseudomallei* and LPS decreased with age (Fig. 1d; *p* < 0.0001). The highest IL-10 response was detected in individuals that were less than 30. Conversely IFN-γ production was not affected (Fig. 1c) by age and remained constant. The reduced IL-10 production in older individuals was not caused by changes in the number of total white blood cells, neutrophils, lymphocytes, monocytes, eosinophils and basophils (Table 1).

### CD3^−^CD14^+^ monocytes are the major cellular source of IL-10 in response to *B. pseudomallei*

**Figure 2:**
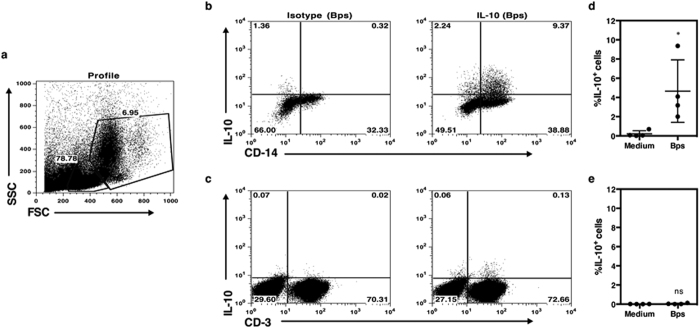
CD3^−^ CD14^+^ monocytes are the main IL-10 producers in response to *B. pseudomallei*. PBMCs from healthy seropositive individuals were incubated with killed *B. pseudomallei* for 20 hours and, intracellular IL-10 versus cell surface expression of either CD3 or CD14 detected by flow cytometry. The profile (**a**) of one representative donor was gated on monocytes (**b**) and lymphocytes (**c**). **d** and **e** shows the frequency of IL-10 producing CD3^−^CD14^+^ monocytes (**d**) or CD3^+^ lymphocytes (**e**) from multiple individuals (n = 4). The data in (**d**) are presented with the mean and the standard deviation. Statistical significance of killed *B. pseudomallei* versus medium alone was determined using paired T test; ns, non significant, *p < 0.05, **p < 0.01 and ***p < 0.001.

To identify the cellular sources of IL-10, PBMCs of seropositive healthy individuals were incubated with *B. pseudomallei* for 20 hours, then subsequently stained for cell surface phenotype markers (CD3 and CD14), and intracellular IL-10 and analyzed by flow cytometry. In a representative donor (Fig. 2a–c), incubation of PBMC resulted in clear induction of total IL-10^+^ cells and the majority was CD14^+^ cells within the monocyte gating area (Fig. 2a,b). In contrast, IL-10 production from CD3^+^ cells within the T cell gate was minimal at 20 hours and not significantly different in the presence of *B. pseudomallei* (Fig. 2c). The predominance of monocytes responding to *B. pseudomallei* for IL-10 production rather than T cells was confirmed in further 3 healthy individuals (Fig. 2d,e).

### Neutralization of IL-10 increased IFN-γ and TNF-α production to *B. pseudomallei*

**Figure 3:**
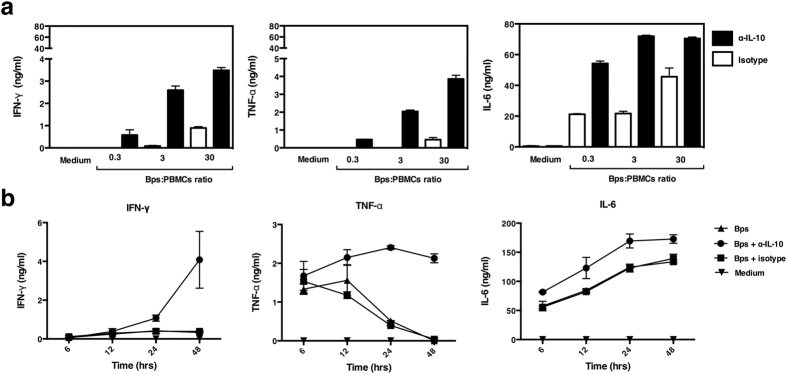
Neutralization of IL-10 increases IFN-γ, TNF-α and IL-6 production in response to *B. pseudomallei.* PBMCs of one representative donor were stimulated with killed *B. pseudomallei* at various ratios (0.3:1, 3:1 or 30:1) with or without the addition of anti-IL-10 mAb for 48 hours *in vitro* (**a**). Kinetics of PMBCs cytokine responses to killed *B. pseudomallei* (30:1 ratio) in the presence or absence of anti-IL-10 mAb for 6, 12, 24 or 48 hours *in vitro* (**b**). IFN-γ, TNF-α and IL-6 in cell supernatants were measured by ELISA. The data are presented with the mean and the standard deviation.

**Figure 4:**
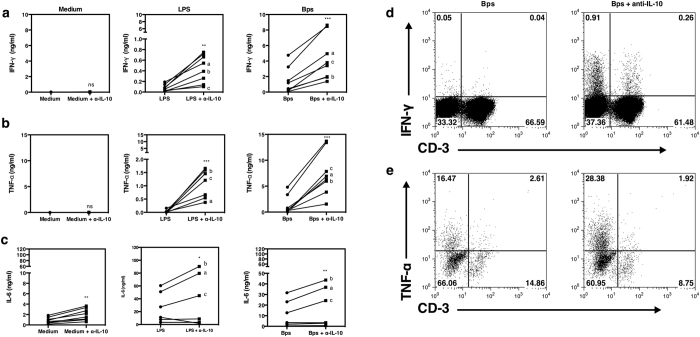
Neutralization of IL-10 increases IFN-γ and TNF-α production by CD3^+^ versus CD3^−^ cells in response to *B. pseudomallei.* PBMCs of healthy seropositive individuals (n = 8) were incubated with 10 μg/ml *E. coli* LPS or killed *B. pseudomallei* (30:1 ratio) versus medium alone and in the presence or absence of 3 μg/ml of anti-IL-10 mAb. IFN-γ (**a**), TNF-α (**b**) and IL-6 (**c**) production was measured by ELISA from collected supernatant after 48 hours *in vitro*. Each symbol represents data from an individual. The values from the same individual in the presence or absence of anti-IL-10 mAb condition are joined by a line. To identify the cellular source of cytokines, PBMCs were incubated with killed *B. pseudomallei* (30:1 ratio) for 20 hours and assayed by flow cytometry for intracellular IFN-γ and TNF-α versus cell surface expression of CD3. A profile of one representative donor, gated initially on lymphocytes (**d**) and monocytes (**e**) by FSC/SSC shows the frequency of IFN-γ and TNF-α producing cells respectively. Statistical significance was determined using paired T test; ns, non significant, *p < 0.05, **p < 0.01 and ***p < 0.001. IL-6 statistical significance was determined from 3 out of 6 responding donors.

To assess the potential immunoregulatory effect of IL-10 on immune responses to *B. pseudomallei,* we first added exogenous recombinant human IL-10 to the cytokine induction assays described above. We observed dose dependent inhibition of the IFN-γ response, with complete inhibition at 1ng/ml of IL-10 added (data not shown). To examine the effect of *B. pseudomallei* induced IL-10 in regulating pro-inflammatory cytokine production, neutralizing anti-IL-10 was then added to PBMCs from healthy individuals in the presence of killed bacteria. Addition of anti-IL-10 monoclonal antibodies (mAb) increased IFN-γ, TNF-α and IL-6 production in a dose dependent manner (not shown) and was also observed in all *B. pseudomallei* to PBMCs ratios (Fig. 3a). Increased cytokine production was greatest at 48 hours but the kinetics of the effects differed according to the cytokine tested (Fig. 3b). Neutralization of IL-10 increased IFN-γ production predominantly at 48 hours, extended the total duration of the TNF-α response and increased IL-6 production at all time points. Purified monocytes also showed elevated TNF-α and IL-6 in response to *B. pseudomallei* at the same time points in the presence of anti-IL-10 mAb (Supplementary Fig. S1). In all 8 donors the neutralization of IL-10 also increased IFN-γ and TNF-α cytokine production (Fig. 4a,b). However IL-6 production only increased when IL-10 was neutralized in responding donors (Fig. 4c). To determine the cellular sources of the pro-inflammatory cytokines, PBMCs were stimulated for 24 hours with *B. pseudomallei* in the presence or absence of anti-IL-10 mAb; the surface markers of PBMCs were stained for CD3, TNF-α and IFN-γ. Flow cytometry gated on the lymphocyte population of a representative donor (Fig. 4d) showed that IL-10 neutralization increased the frequency of IFN-γ positive CD3^−^ cells from 0.05% to 0.91% and T cells (CD3^+^) from 0.04% to 0.26%. The frequency of TNF-α positive cells from the CD3^−^ monocyte population (Fig. 4e) also increased from 16.47% to 28.38%. Taken together the findings show a trend that IL-10 produced in response to *B. pseudomallei* may suppress the ability of monocytes, NK cells and T cells to produce pro-inflammatory cytokines.

### Neutralization of IL-10 also enhances pro-inflammatory cytokine responses in individuals with DM

**Figure 5:**
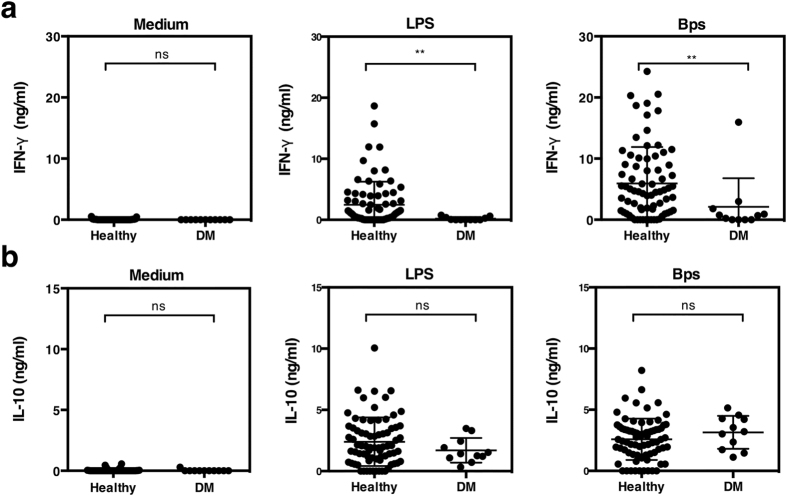
IFN-γ production in response to *B. pseudomallei* is reduced in individuals with DM. Whole blood of healthy seropositive individuals (n = 75) and individuals with DM (n = 11) were incubated with killed *B. pseudomallei* (30:1 ratio), 10 μg/ml of *E. coli* LPS or medium alone and culture supernatants assayed for IFN-γ (**a**) and IL-10 (**b**) by ELISA after 48 hours *in vitro*. The data are presented with the mean and the standard deviation. Statistical significance was determined by Mann-Whitney test; ns, non significant, *p < 0.05, **p < 0.01 and ***p < 0.001.

**Figure 6:**
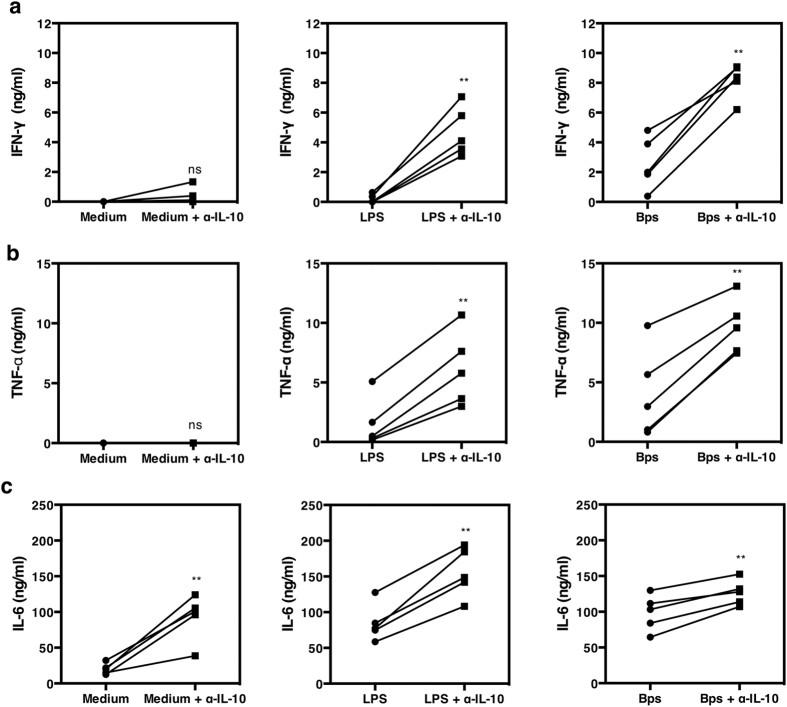
Neutralization of IL-10 increases IFN-γ, TNF-α and IL-6 production in individuals with DM. PBMCs (n = 5) were incubated with 10 μg/ml *E. coli* LPS, killed *B. pseudomallei* (30:1 ratio) or medium alone in the presence or absence of anti-IL-10 mAb. IFN-γ (**a**), TNF-α (**b**) and IL-6 (**c**) was measured by ELISA from collected supernatant after 48 hours *in vitro*. Each symbol represents data from an individual. The values from the same individual in the presence or absence of anti-IL-10 mAb are joined by a line. Statistical significance was determined using paired T test; ns, non significant, *p < 0.05, **p < 0.01 and ***p < 0.001.

A previous study found individuals with DM in Singapore to have impaired IL-12 production that resulted in reduced IFN-γ responses to *B. pseudomallei* by PBMCs *in vitro*^7^. To determine whether individuals with DM in northeast Thailand also had altered cytokine responses to *B. pseudomallei*, the production of IFN-γ and IL-10 from healthy individuals and individuals with DM was compared (Supplementary Table S1). Whole blood was incubated with killed *B. pseudomallei* for 48 hours and IL-10 and IFN-γ production was determined by ELISA. In response to either *B. pseudomallei* or LPS, IFN-γ production was reduced in individuals with DM (Fig. 5a; *p* < 0.01) but IL-10 production was unimpaired in response to *B. pseudomallei* (Fig. 5b). The difference in IFN-γ responses between healthy individuals and individuals with DM was also observed when the individuals were also age matched, suggesting that blood glucose status and not age was the primary determinant (data not shown). The baseline of IL-6 production was high in individuals with DM when compared to healthy individuals in our PBMCs culture (Figs 4c and 6c). Neutralization of IL-10 in PBMCs from individuals with DM increased IFN-γ, TNF-α and IL-6 production (Fig. 6a–c). In particular, neutralization of IL-10 in individuals with DM restored IFN-γ responses to levels observed in healthy individuals. These observations suggest that the IL-10 is at least in part responsible for the impaired *in vitro* IFN-γ responses seen in these individuals who are most at risk of infection.

### Neutralization of IL-10 improves phagocyte-mediated killing of *B. pseudomallei* in both healthy individuals and individuals with DM

**Figure 7:**
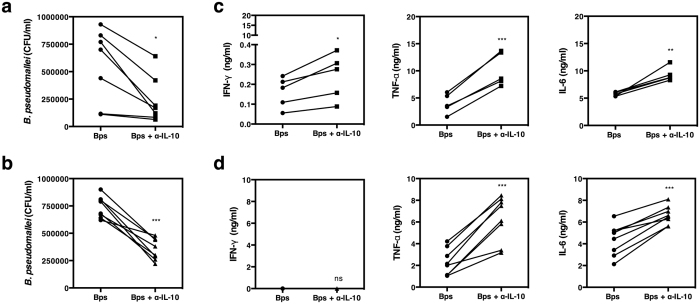
Neutralization of IL-10 increases the killing of *B. pseudomallei* by healthy individuals and individuals with DM. PBMCs from healthy individuals (n = 7) (**a**) and individuals with DM (n = 8) (**b**) were incubated with medium, live *B. pseudomallei* at an MOI of 1 in the presence or absence of 3 ug/ml of anti-IL-10 mAb for 6 hours. The number of live *B. pseudomallei* was assessed by colony forming unit assay. The cultured supernatants of healthy individuals (n = 5) (**c**) and individuals with DM (n = 8) (**d**) were assayed for IFN-γ, TNF-α and IL-6 by ELISA. Each symbol represents data from an individual. The values from the same individual in the presence or absence of anti-IL-10 mAb are joined by a line. Statistical significance was determined using paired T test; ns, non significant, *p < 0.05, **p < 0.01 and ***p < 0.001. Of note, cultured supernatants from 5 (**c**) out of 7 (**a**) healthy individuals were sufficient to be analyzed for cytokine response.

To ask whether IL-10 could interfere with the microbicidal activity of host cells to kill *B. pseudomallei*, PBMCs from healthy individuals and individuals with DM were infected with living *B. pseudomallei* in the presence or absence of anti-IL-10 mAb and the number of viable bacteria determined 24 hours later. Improved bacterial killing was observed in PBMCs treated with anti-IL-10 prior to infection with *B. pseudomallei* in both healthy individuals and individuals with DM (Fig. 7a; *p* < 0.05 and Fig. 7b; *p* < 0.001 respectively), compared to those without IL-10 neutralization. This was also observed in isolated monocytes (Supplementary Fig. S2; *p* < 0.01). In healthy individuals, the increased killing was associated with elevated production of IFN-γ, TNF-α and IL-6 in the presence of anti-IL-10 mAb only 6 hours after infection (Fig. 7c; *p* < 0.05, <0.001, <0.01 respectively). In individuals with DM (Fig. 7d; *p* < 0.001) neutralization of IL-10 increased TNF-α and IL-6 cytokine production but at this time point IFN-γ was not detected either in the presence or absence of IL-10 neutralization. Together, these results demonstrate that *B. pseudomallei* is a potent inducer of IL-10 production, which acts to regulate production of pro-inflammatory cytokines but also reduces the microbicidal activity of host cells against this pathogen *in vitro*.

## Discussion

IL-10 is a potent immunomodulatory cytokine found in the serum of individuals with acute melioidosis, but until now its cellular source, impact on host immune functions and relationship to the increased susceptibility of individuals with DM to melioidosis was not known. In this study, we have shown that incubation of intact *B. pseudomallei* with whole blood of healthy individuals living in the melioidosis endemic region of northeastern Thailand induces both IFN-γ and IL-10. In contrast, this balance is skewed in favor of IL-10 production in individuals with DM who are most at risk of infection. The functional consequences of this monocyte derived IL-10 response in both donor groups is to impair production of the pro-inflammatory cytokines IFN-γ, TNF-α and IL-6, and to reduce the killing of *B. pseudomallei* by host phagocytes.

Acute presentation with signs of pneumonia, bacteremia and septic shock are the most common and serious manifestations of human melioidosis^5^. Analysis of serum cytokine expression in these patients has shown increased concentrations of both pro-inflammatory cytokines, including IFN-γ, TNF-α, IL-6 and the immunoregulatory cytokine IL-10^21^,^22^, with expression of the latter two cytokines an independent predictor of mortality^22^. Here, we found that incubation of human peripheral blood from healthy seropositive individuals from the melioidosis endemic region of N.E. Thailand resulted in the increased expression of the same cytokines in a dose and time dependent manner *in vitro*. The magnitude of our response was similar to that observed with other established inducers of IL-10 secretion, and is consistent with previous reports of immune cell activation by either live or killed whole *B. pseudomallei* or *B. pseudomallei* derived LPS via a TLR-dependent process^3^,^32^,^33^,^34^. The magnitude of response in human samples may vary between individuals however we were able to show a similar trend in cytokine response in this population. However some studies in mice infected with *B. pseudomallei* have found no increase in IL-10 production^14^,^35^ whereas other studies using live *B. pseudomallei*^18^ or earlier time points^12^ were able to detect IL-10 along with other pro-inflammatory cytokines in response to infection.

Previous studies using other intracellular bacteria such as *L. monocytogenes* and *M. tuberculosis*, which share some common features in intracellular location and pathology with *B. pseudomallei,* found marginal zone B cells and macrophages to be the major sources of IL-10 respectively. Here, flow cytometric analysis of human PBMCs incubated with live *B. pseudomallei* showed CD3^−^CD14^+^ monocytes to be the main IL-10 producing cells from 6 to 20 hours of culture. In contrast, CD3^+^ T cells were not a substantial source of IL-10 under these conditions, despite the fact that all blood donors had evidence of adaptive immune responses to *B. pseudomallei* antigens by serology and the presence of antigen specific, IFN-γ secreting T cells at this time^17^. The magnitude in response in this low cohort of samples was able to significantly identify the main cellular source of IL-10. However additional samples may be added to confirm this finding. Nonetheless our result is consistent with our evidence to date that natural exposure to *B. pseudomallei* primarily induces a Th1 oriented T cell response, and suggests there is little or no expression of Th2, IL-10^+^ Treg or dual IFN-γ^+^+/IL-10^+^ T cells in these individuals.

In other systems, IL-10 is known to inhibit many cell mediated immune responses which are involved in protection against intracellular pathogens^23^,^36^,^37^. IL-10 suppresses macrophage and dendritic cell (DC) functions, including intracellular pathogen killing and production of IL-12, TNF-α and IFN-γ required for effective Th1 responses^38^, each of which have been shown to be involved in immunity to *B. pseudomallei* either in animal models or in humans. Here, direct addition of rIL-10 to human PBMCs in the presence of *B. pseudomallei* reduced production of IFN-γ in a dose dependent manner, with complete inhibition of this response at IL-10 concentrations, which were found both in cell culture and in the serum of infected patients. Furthermore, neutralizing endogenous IL-10 in co-cultures of PBMCs with *B. pseudomallei* increased the production of IFN-γ, TNF-α and IL-6 in the majority of individuals tested. Thus, IL-10 produced during the innate immune response to *B. pseudomallei* actively regulates the pro-inflammatory cytokine response of the host. TNF-α is an important regulator of cell migration and inflammation in other bacterial infections, and mice depleted of TNF-α by neutralizing mAb *in vivo* have increased susceptibility to *B. pseudomallei*^11^. The direct contribution of IL-6 in melioidosis is not known but in addition to being a key component of the acute phase response, IL-6 can regulate recruitment and stimulation of neutrophils^39^, which are a crucial first line of defense during melioidosis^9^. Perhaps most importantly for control of bacterial replication, the IFN-γ response to *B. pseudomallei* seems to be particularly sensitive to down regulation by endogenous IL-10. IFN-γ is the primary cytokine responsible for activation of macrophages for the respiratory burst oxygen dependent killing of *B. pseudomallei* by macrophages^40^. Both NK cells and CD3^+^ T cells are important sources of IFN-γ in mice and humans following exposure to *B. pseudomallei*, and IFN-γ KO mice are exquisitely susceptibly to experimental melioidosis^11^,^17^. Here, IL-10 depletion *in vitro* increased IFN-γ production by both NK cells and CD3^+^ T cells, leading to a 10-fold increase in total IFN-γ production with more rapid kinetics allowing significant production of IFN-γ by 24 hours of culture. Furthermore, neutralizing IL-10 in mice has also shown an increased in IFN-γ response^18^. Importantly, neutralization of IL-10 additionally improved the killing of the bacterium by PBMCs and monocytes suggesting that these antimicrobial responses are actively down regulated by IL-10 under these conditions. Finally, IL-10 may also have other functional effects on the immune response to *B. pseudomallei* such as expression of type I IFNs, shown to interfere with cytokine production and increase susceptibility to other bacterial infections^41^ and in the induction of program death ligand 1 (PD-L1) expression^42^.

In melioidosis endemic regions, the incidence of acute infection is greatest in older individuals and in particular those with poorly controlled DM. In N.E. Thailand where our study was sited, melioidosis has been reported to be highest in the 55–64 age group^43^,^44^. We found IL-10 production decreased with age in response to *B. pseudomallei* and the TLR4 ligand, LPS. In contrast there was no association between age and production of IFN-γ in the same population. The immune responses of aging populations have mostly been studied in the context of viral infections, showing a heightened pro-inflammatory cytokine background in older individuals, which is suggested to lead to increased tissue damage^31^. Our study with the bacterial immune activators LPS and intact *B. pseudomallei* supports this possibility, although further studies on the susceptibility to melioidosis with age, independent of the confounding effect of DM in these populations, is needed.

Diabetes mellitus (DM) is the strongest predisposing risk factor for developing acute melioidosis^6^,^45^. Multiple immune defects have been described in individual with poor glycaemic control which could contribute to this susceptibly, including impaired immune function especially in neutrophils, which includes phagocytosis and the oxidative burst^9^. A key study in Singapore reported that type 2 diabetics had a deficiency in intracellular glutathione (GSH), which impaired production of both IL-12 and IFN-γ that led to poor control of *B. pseudomallei* replication^7^. Here we also observed reduced IFN-γ responses of PBMCs of Thai individuals with DM to both LPS and *B. pseudomallei*, suggesting that impairment of IFN-γ production to bacterial components is indeed a common feature of DM and may contribute to their susceptibility to a broad range of pathogens that require IFN-γ activated macrophages for their elimination^46^. In contrast, IL-10 responses were not impaired, resulting in an altered ratio of pro-inflammatory versus anti-inflammatory cytokine production. Importantly, neutralization of IL-10 in individuals with DM restored the reduced IFN-γ production to levels similar to the healthy individuals and increased the killing of *B. pseudomallei in vitro*. Although we did not detect IL-12 directly under these culture conditions, it is likely that this is mediated by changes in IL-12 since IFN-γ responses to *B. pseudomallei* are IL-12 dependent^47^, and IL-10 is well recognized to interfere with IL-12p35 and p40 production^48^,^49^. In summary, although the numbers of donors tested was not large in all assays, production of IL-10 in response to *B. pseudomallei* and increased pro-inflammatory cytokine production and bacterial killing were observed in the majority of individuals tested (66 out of 75 for production of IL-10, 5 out of 5 for increased IFN-γ and TNF-α secretion and 5 out of 7 donors tested for bacterial killing). Future studies using larger sample sizes and assaying the effect of IL-10 neutralization on other immune functions, such as CD8^+^ T cells, would further strengthen our understanding of the role of IL-10 in melioidosis.

In conclusion, we have shown that production of the immunoregulatory cytokine IL-10 is a prominent feature of the human innate immune response to *B. pseudomallei*, and actively inhibits both pro-inflammatory and antimicrobial responses of the host. Inhibition of the antimicrobial actions of IFN-γ, TNF-α and IL-6 are likely to increase the susceptibility of the host to infection. In contrast, IL-10-mediated control of excessive production of TNF-α and IL-6, which drive the pathological effects of the ‘cytokine storm’ found in patients with sepsis and septic shock may on the other hand be beneficial. Individuals with DM are most at risk of developing clinical melioidosis, but in contrast are less likely to die^50^,^51^. Our data showing poor IFN-γ responses but maintenance of IL-10 production in these individuals may help to explain these clinical findings. More detailed information on the effects of DM on pro-inflammatory versus immunoregulatory immune responses against *B. pseudomallei* should provide a better understanding of the pathogenesis of melioidosis and provide a basis for improved treatment and prevention by vaccination.

## Methods

### Subjects

This study utilized peripheral blood obtained from healthy individuals versus individuals with DM obtained following written informed consent, and authorized by the Khon Kaen University Ethics Committee, research number HE470506. The study was carried out in accordance with the approved guidelines and all subjects provided written informed consent. Donors attending the Khon Kaen Medical Centre, were screened for fasting blood sugar levels, blood pressure and their history of infections and hospitalization were determined. Those who had an antibody assay index to crude *B. pseudomallei* extract of 2 or more were considered seropositive, equivalent to an indirect hemagglutination assay (IHA) of more than 40, were included in this study^52^. Measurement of serum antibodies to *B. pseudomallei* was performed with 96 well polystyrene plates (Nunc Maxisorp) either uncoated or coated with 1 μg/ml of *B. pseudomallei* K96243 crude extract in 0.1 M carbonate bicarbonate buffer (pH 9.6) and incubated overnight at 4 °C. Plates were washed and 50 μl/well of 1:300 diluted human plasma were probed in duplicates. Immunoreactivity was detected by using biotinylated-rabbit anti human IgG followed by horseradish peroxidase tagged with streptavidin, after incubation for 1 hour at room temperature. The color was developed with tetramethylbenzidine substrate (BD Biosciences) and the reaction was stopped with 2 N H_2_SO_4_. Optical density (O.D) of each well was read at 450 nm and the results are represented as absorbance index of individual sample = (O.D_test_ − O.D_uncoated_)/O.D_uncoated_. Healthy individuals aged between 17 and 78 years from Khon Kaen in the northeast region of Thailand used in this study were defined with fasting blood sugar of less than 126 mg/dL, normal blood pressure around 120/80 mm/Hg and had no clinical history of melioidosis. Individuals with DM, were defined as diagnosed with type 2 DM by a physician and fasting blood sugar level of more than 126 mg/dL^53^ at the time of blood collection. Blood samples from both groups were collected, processed and assayed in parallel in the compared experiments.

### Cell culture

Whole blood (WB) was collected in heparinized tubes (BD Biosciences). The total number of lymphocytes plus monocytes was determined using an automated machine (Sysmex) and adjusted to 1.8 × 10^6^ cells/ml with R10, which contains RPMI 1640 (Gibco), 10% heat inactivated fetal bovine serum (BioWest), 100 U/ml Penicillin/Streptomycin (Gibco), 50 μg/ml gentamycin (Sigma), and 25 mM HEPES (Gibco). Adjusted WB cells were plated in duplicates onto 96 well plates (Nalgene) and incubated in the presence of *Escherichia coli (E.coli*) lipopolysaccharide (LPS) 10 μg/ml (Sigma) or a 30:1 ratio of killed *B. pseudomallei* to PBMCs concentration. Killed *B. pseudomallei* was prepared from strain K96243 a clinical isolate from Thailand^54^; the whole bacteria were fixed with 2% paraformaldehyde for 1 hour at room temperature, washed twice with 1 × PBS (pH 7.4) and stored at −80 °C. After incubation of WB with stimuli for 48 hours at 37 °C in 5% CO_2_, supernatant was collected and stored at −80 °C for cytokine measurement by ELISA^55^.

In other experiments, human PBMCs were separated by density gradient centrifugation using Ficoll-Paque (BioWest). The PBMCs were collected and washed twice with 1 × PBS and re-suspended in R10. Cells were seeded at 5 × 10^6^ cells/ml into a 96 well plate and incubated in the presence of the same conditions as the whole blood assay either with or without 3 μg/ml of neutralizing anti-human IL-10 (anti-IL-10) monoclonal antibodies (mAb), clone JES3-19F1 (BD Biosciences) or its isotype control, clone R35-95.

Primary human monocytes were isolated from recently separated human PBMCs by positive selection using magnetic CD14 microbeads, according to the manufacturers instructions (Miltenyi Biotec). The CD14^+^ cells were obtained and washed with 2 mM of EDTA in 1 × PBS and re-suspended in R10. Cells were seeded at 5 × 10^5^ cells/ml into 96 well plates. Primary monocytes were treated with the same conditions as the PBMCs.

The concentration of IFN-γ, IL-10, IL-6 and TNF-α in supernatants from WB, PBMCs or isolated primary monocytes were measured with commercial ELISA kits (BD Biosciences) according to the manufacturers instructions.

### *B. pseudomallei* killing assay

*B. pseudomallei* strain K96243, was grown to mid-log phase at 37 °C in Luria-Bertani (LB) broth and assessed by optical density at 600 nm; an absorbance index of 1 was equivalent to 10^9^ CFU/ml of bacteria. The PBMCs or isolated primary monocytes were treated with 10 μg/ml of anti-IL-10 mAb for 15 minutes prior to infection with *B. pseudomallei* at multiplicity of infection (MOI) of 1 and incubated at 37 °C for 6 hours. The supernatant was collected for cytokine analysis and the infected cells were lysed by 1% Triton X-100 (Biotech). The bacterial colony count was determined by standard bacterial plating on LB agar plates after 24 hours.

### Flow cytometry for detection of intracellular cytokines

PBMCs were stimulated for 2 hours before 3 μg/ml Brefeldin A (Sigma Aldrich) was added; surface and intracellular cytokine staining was performed 18 hours later. Cell surface phenotype was determined by incubating with the following mAb: anti-CD3 PerCP (clone UCHT1), anti-CD4 APC (clone OKT4), anti-CD14 FITC (clone MΦP9) (BD Biosciences). Intracellular cytokine production was detected with anti-IFN-γ FITC (clone 4SB3), anti-TNF-α PE (clone MAb11) (BioLegend) and anti-IL-10 PE (clone JES3-9D7) (BD Biosciences). Isotype matched control antibodies were used in each analysis. Data were acquired using a FACS Caliber flow cytometer and analyzed using FlowJo version 9.3.2 (Tree Star) with 50,000 cells acquired from either the monocytes or the lymphocytes population.

### Data analysis

All data were analyzed for statistical significance using Prism 5 software (GraphPad) using either one way ANOVA, Tukey’s post test, Pearson correlation, paired T and Mann-Whitney test as appropriate to the experimental design and specified in the figure legends. P values of ≤0.05 were considered statistically significant.

## Additional Information

**How to cite this article**: Kessler, B. *et al*. Interleukin 10 inhibits pro-inflammatory cytokine responses and killing of *Burkholderia pseudomallei. Sci. Rep.* **7**, 42791; doi: 10.1038/srep42791 (2017).

**Publisher's note:** Springer Nature remains neutral with regard to jurisdictional claims in published maps and institutional affiliations.

## Supplementary Material

Supplementary Information

## Figures and Tables

**Table 1:** Demographic data of healthy individuals whole blood screening.

Age groups	<35	35–64	>64	P value
Number of subjects	16	37	22	—
Mean age	25.5 (4.9)	49.2 (6.3)	69.8 (4.1)	—
Sex (Male/Female)	6/10	6/31	4/18	Δ
WBC	7.4 (2.1)	6.1 (1.5)	6.0 (1.6)	ns
Neutrophils	1.4 (4.4)	3.1 (1.0)	3.1 (0.8)	*
Lymphocytes	2.3 (0.6)	2.4 (0.7)	2.1 (0.8)	ns
Monocytes	0.4 (0.1)	0.3 (0.1)	0.3 (0.1)	ns
Eosinophils	0.2 (0.3)	0.3 (0.2)	0.5 (0.4)	*
Basophils	0.03 (0.01)	0.03 (0.02)	0.03 (0.02)	ns

WBC and sub-type measures are reported in cells ×10^6^/ml [mean (SD)].

SD = standard deviation.

Δ No effect of gender on IL-10 and IFN-γ response to *B. pseudomallei* and controls.

Statistical significance was determined using one-way ANOVA; ns, non significant.

^*^p < 0.05.
