# Supplementary material for: Interleukin 10 inhibits pro-inflammatory cytokine responses and killing of Burkholderia pseudomallei
Source: Sci Rep. 2017 Feb 20;7:42791. doi: 10.1038/srep42791 (PMC5316963; doi:10.1038/srep42791)
Supplement: Supplementary Information [file srep42791-s1.pdf]

Interleukin 10 inhibits pro-inflammatory cytokine responses and killing of *Burkholderia pseudomallei*

Bianca Kessler[1], Darawan Rinchai[1,4], Chidchamai Kewcharoenwong[1], Arnone Nithichanon[1], Rachael Biggart[2], Catherine M. Hawrylowicz[3], Gregory J. Bancroft[2*], Ganjana Lertmemongkolchai[1*]

[1]Centre for Research and Development of Medical Diagnostic Laboratories, Faculty of Associated Medical Sciences, Khon Kaen University, Khon Kaen, Thailand.

[2]Department of Immunology and Infection, London School of Hygiene & Tropical Medicine, London, United Kingdom.

[3]Department of Asthma, Allergy and Respiratory Science, Guys Hospital, Kings College, London, United Kingdom.

***To whom correspondence should be addressed**

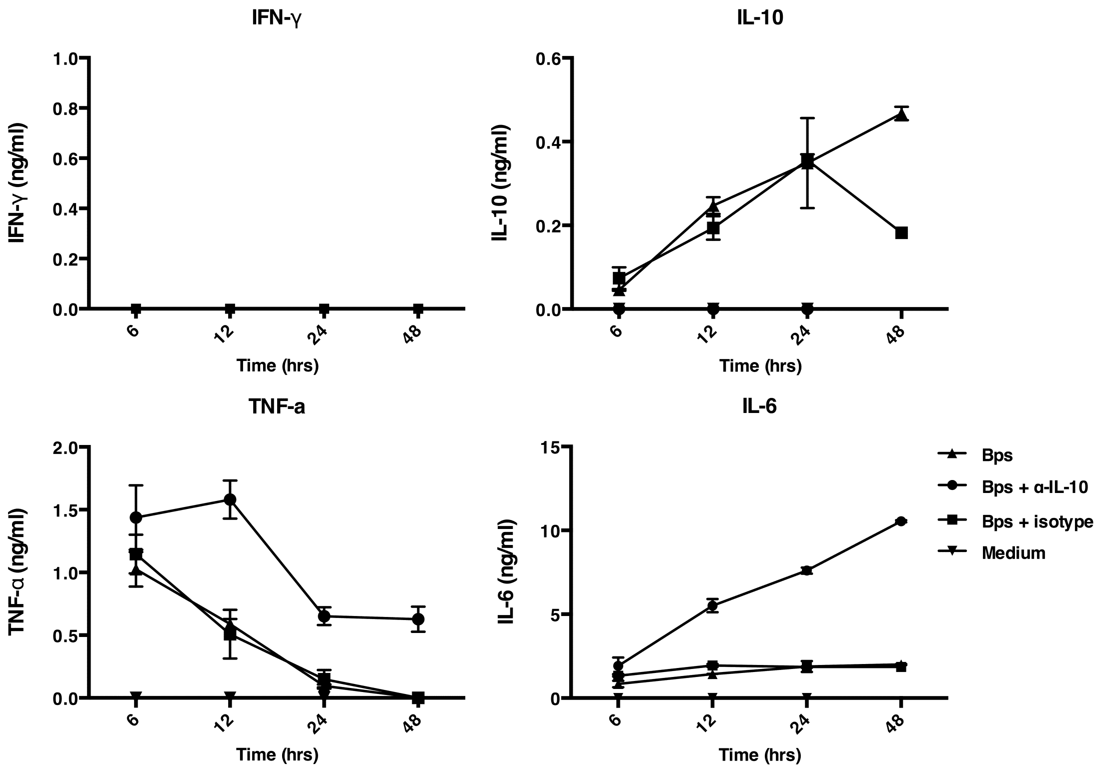

Supplementary Figure S1: Kinetics of CD14$^+$ monocytes cytokine secretion in response to *B. pseudomallei*. Purified CD14$^+$ monocytes were incubated with killed *B. pseudomallei* (30:1 ratio) in the presence or absence of anti-IL-10 mAb for 6, 12, 24 or 48 hours *in vitro* and culture supernatants assayed for IFN-γ, IL-10, TNF-α and IL-6 by ELISA.

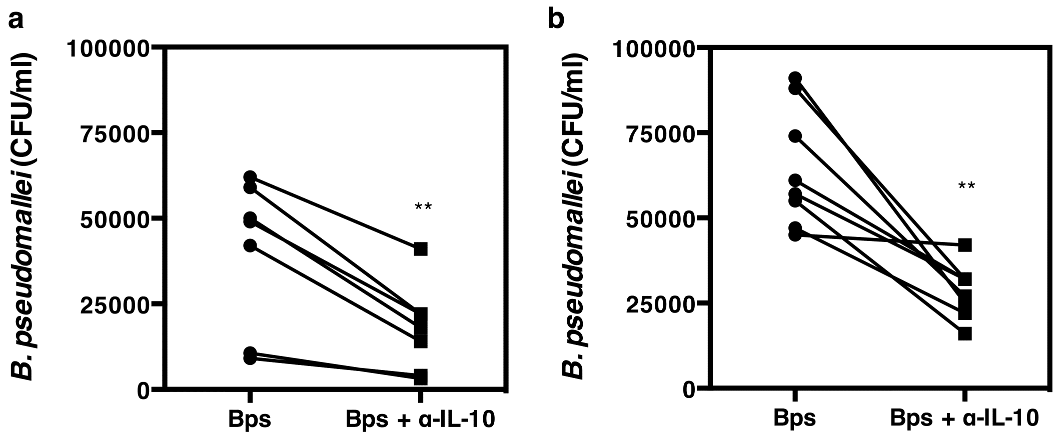

Supplementary Figure S2: Neutralization of IL-10 increases the killing of *B. pseudomallei* by CD14$^+$ monocytes. Monocytes from a healthy seropositive representative were incubated with medium, alone or live *B. pseudomallei* at an MOI of 1 in the presence or absence of 3μg/ml of anti-IL-10 mAb for 6 hours. The number of live *B. pseudomallei* was assessed by colony forming unit assay in healthy individuals (n=5) (**a**) and individuals with DM (n=8) (**b**). Statistical significance was determined using paired T test; ns, non significant, * $p < 0.05$, ** $p < 0.01$ and *** $p < 0.001$.

Supplementary Table S1: Demographic of healthy individuals and individuals with DM whole blood

	**Healthy**	**Diabetes Mellitus**
Number of subjects	75	11
Mean age (SD)	47.9 (22)	56.5(14)
Sex (Male/Female)	16/59	2/5
Fasting Blood glucose, mg/dL (SD)	102 (12)	188 (14)
WBC (SD)	6.5 (1.7)	7.4 (2.2)
Neutrophils (SD)	2.5 (2.0)	4.3 (2.6)
Lymphocytes (SD)	2.3 (0.7)	2.4 (0.8)
Monocytes (SD)	0.3 (0.1)	0.4 (0.2)
Eosinophils (SD)	0.3 (0.3)	0.3 (0.2)
Basophils (SD)	0.03 (0.02)	0.05 (0.02)

SD = standard deviation
